# Efficacy of the ABC Pathway for Integrated Care Across Phenotypes of Patients with Atrial Fibrillation: A Latent-Class Analysis Report from the mAFA-II Clinical Trial

**DOI:** 10.1007/s11606-024-09037-6

**Published:** 2024-10-28

**Authors:** Bernadette Corica, Giulio Francesco Romiti, Davide Antonio Mei, Marco Proietti, Hui Zhang, Yutao Guo, Gregory Y. H. Lip, on behalf of the mAF-App II trial investigators

**Affiliations:** 1 Liverpool Centre for Cardiovascular Sciences at University of Liverpool, Liverpool John Moores University of Liverpool Heart & Chest Hospital, Liverpool, UK; 2 Department of Translational and Precision Medicine, Sapienza – University of Rome, Rome, Italy; 3 Cardiology Division, Department of Biomedical, Metabolic and Neural Sciences, University of Modena and Reggio Emilia, Policlinico Di Modena, Modena, Italy; 4 Department of Clinical Sciences and Community Health, University of Milan, Milan, Italy; 5 Geriatric Unit, IRCCS Istituti Clinici Scientifici Maugeri, Milan, Italy; 6 Department of Pulmonary Vessel and Thrombotic Disease, Medical School of Chinese PLA, Chinese PLA General Hospital, Beijing, China; 7 Danish Center for Health Services Research, Department of Clinical Medicine, Aalborg University, Aalborg, Denmark

**Keywords:** atrial fibrillation, integrated care, latent class analysis, prognosis

## Abstract

**Background:**

The mAFA-II cluster randomised trial demonstrated the efficacy of a mobile health-technology implemented ‘Atrial fibrillation Better Care’ (ABC) pathway (mAFA intervention) for integrated care management of patients with AF.

**Objective:**

To evaluate the effect of mAFA intervention across phenotypes of patients with AF.

**Design:**

We conducted a latent-class analysis (LCA) according to eight variables, including age and comorbidities.

**Participants:**

The mAFA-II trial enrolled AF patients between June 2018 and August 2019 across 40 centres in China.

**Main Measures:**

We evaluated the interaction between the groups identified through LCA, and the effect of mAFA intervention on the risk of the primary composite outcome of all-cause death, stroke/thromboembolism, and rehospitalisations. Results were expressed as adjusted hazard ratio (aHR) and 95% confidence intervals (95% CI).

**Key Results:**

Across the 3324 patients included in the trial (mean age 68.5 ± 13.9 years, 38.0% females), we identified three phenotypes: (i) low morbidity phenotype (*n* = 1234, 37.1%), (ii) hypertensive/coronary artery disease (CAD) phenotype (*n* = 1534, 46.2%), and (iii) mixed morbidity phenotype (*n* = 556, 16.7%). The effect of mAFA intervention on the primary outcome appeared greater in the low morbidity phenotype (aHR, 0.08; 95% CI 0.02–0.33) compared to the hypertensive/CAD (aHR, 0.30; 95% CI 0.16–0.58) and the mixed morbidity phenotype (aHR, 0.68; 95% CI 0.37–1.24), with a statistically significant interaction (*p*_int_ = 0.004).

**Conclusions:**

In patients with AF, the ABC pathway improved prognosis across different comorbidity phenotypes, although with some differences in the magnitude of risk reduction. Patients with more complex phenotypes require further efforts to improve their outcomes, considering their high baseline risk of adverse events.

**Trial Registration:**

WHO International Clinical Trials Registry Platform (ICTRP) Registration number: ChiCTR-OOC-17014138.

**Supplementary Information:**

The online version contains supplementary material available at 10.1007/s11606-024-09037-6.

## INTRODUCTION

Non-communicable chronic conditions are the leading cause of morbidity, disability and mortality worldwide.^1^ The coexistence of different comorbidities, entailing the so-called multimorbidity, is common in elderly people, and the impact on outcomes might change when two or more conditions are present.^2^ Atrial fibrillation (AF), the commonest arrhythmia, rarely occurs isolated, being often found with other conditions at the time of diagnosis.^3,4^ Specifically, comorbidities in patients with AF tend to cluster, determining different patterns of risk depending on the number and on the type of associated illnesses, multimorbidity and polypharmacy, leading to clinically complex phenotypes that have implications for their treatment and outcomes.^5–9^ Indeed, common comorbidities that increase the outcomes related to the thromboembolic and bleeding risks of AF are included clinical risk scores to aid risk stratification.^10–12^

While multimorbidity has been previously associated with lower use of an oral anticoagulant (OAC)^3^ and an overall worse prognosis of patients with AF, prior research has tried to identify clusters of AF patients according to their risk factors and comorbidities, showing how different risk profiles are associated with heterogeneous—yet often unsatisfactory—management of patients with AF.^13–15^

Acknowledging the unmet needs of such clinically complex patients with AF, the ‘Atrial fibrillation Better Care’ (ABC) pathway was proposed as an holistic and integrated care management approach for these patients.^16^ This was tested in the prospective Mobile Health (mHealth) Technology to Improve Care for Patients with Atrial Fibrillation (mAFA-II) cluster randomised trial which showed that a mHealth-implemented ABC pathway improved prognosis in over 3300 patients with AF,^17^ and a subsequent post-hoc analysis confirmed these findings in patients with multimorbidity.^18^ Given the improved clinical outcomes seen with ABC pathway adherence,^19–21^ this approach is now recommended in guidelines.^22^ Nonetheless, whether the effect of the ABC pathway is consistent among patients with different comorbidity phenotypes remains unclear. In this ancillary analysis from the mAFA-II trial, we aimed to explore the effect of mAFA intervention across different phenotypes of AF patients, as defined by their baseline risk factors, using a latent class analysis (LCA) approach.

## METHODS

### Study Design and Procedures

Details on the design, sample size calculation, protocol and primary results of the mAFA-II trial have been previously published and reported.^17,23^ Briefly, the mAFA-II was a cluster-randomised, multicentre and prospective trial that enrolled AF patients ≥ 18 years old across 40 centres in China between June 1, 2018, and August 16, 2019. Twenty centres were randomised to the mAFA intervention and 20 centres to usual care. Patients were excluded if any of these criteria was met: (i) presence of mechanical prosthetic valves, (ii) moderate to severe mitral stenosis, (iii) unable to complete 1-year of follow-up for any reason. Finally, 1646 subjects with AF were allocated to mAFA intervention, while 1678 were managed according to local practice (i.e., ‘usual care’). The study was approved by the Central Medical Ethic Committee of Chinese PLA General Hospital and by local institutional review boards and was conducted in accordance with the Declaration of Helsinki and the Consolidated Standards of Reporting Trials (CONSORT) reporting guideline; all the patients enrolled gave their written informed consent.

Full details on the procedures followed in the study can be found elsewhere.^17,23^ Briefly, in patients allocated to the mAFA intervention group, the ABC pathway was implemented through a user-friendly application for smartphones (one designed for doctors and one for patients), and streamlined as follows: ‘A’ criterion—prescription of OAC based on regular re-assessment of thromboembolic and haemorrhagic risk, with dose adjustments according to renal and liver function tests; ‘B’ criterion—management of symptoms (which included antiarrhythmics and rhythm control therapies) and regular monitoring of patient-reported symptoms (evaluated according to the European Heart Rhythm Association classification); ‘C’ criterion—active management and optimisation of treatment of concurrent comorbidities (e.g. blood pressure monitoring to achieve optimal hypertension management).^14,16^ Patients allocated to ‘usual care’ were managed according to local practices.

### Outcomes and Follow-up

Patients enrolled in the trial were followed up at 6 and 12 months after the inclusion for the occurrence of clinical adverse events. Consistently with the primary trial’s analysis, our primary outcome was the composite of ischemic stroke (IS) or systemic thromboembolism (TE), all-cause death, and rehospitalisation. In this analysis, we also evaluated other secondary exploratory outcomes: all-cause death, thromboembolic and bleeding events, rehospitalisations and the composite of recurrent AF, HF and acute coronary syndrome. For each outcome, we evaluated the effect of mAFA intervention compared to usual care according to class allocation.

### Statistical Analysis

We performed a LCA according to eight variables (including age over 75, coronary artery disease (CAD), HF, renal dysfunction, diabetes mellitus (DM), arterial hypertension, peripheral artery disease (PAD) and prior IS evaluated at baseline.

We selected these variables considering their association with the risk of adverse outcomes, the data availability, the prevalence of the conditions in our cohort and the balance and representativeness of cardiovascular and non-cardiovascular comorbidities.

Data regarding these conditions were recorded by the investigators at baseline in the case report form and were used accordingly for this analysis. The optimal number of latent classes was determined considering a number of criteria, including the Bayesian Information Criterion (BIC), the consistent Akaike Information Criterion (cAIC), the size of the smallest class^24–26^ and also according to clinical judgement. Each patient was then assigned to a class, according to the modal posterior probability.^26^

Baseline characteristics for the primary analysis population were reported as mean and standard deviation (SD) for normally distributed continuous variables, or median and interquartile range (IQR) for non-normally distributed continuous variables. Frequency and percentage were reported for binary or categorical variables.

Multiple-adjusted Cox regression models were used to evaluate the risk of primary and secondary outcomes according to class allocation, and to analyse the interaction between the effect of mAFA intervention and class allocation on the risk of outcomes. Results were expressed as adjusted hazard ratio (aHR) and 95% confidence intervals (95% CI). For the association between phenotypes and the risk of outcomes, we included age, sex, type of AF, allocation to mAFA intervention and cluster effect among the covariates. For the interaction between mAFA intervention and phenotypes on the risk of outcomes, we included age, sex, type of AF, arterial hypertension, DM, CAD, HF, renal dysfunction, history of IS, PAD and cluster effect among the covariates. A two-sided *p*-value < 0.05 was considered statistically significant. All statistical analyses were conducted with R 4.2.0 (R Foundation for Statistical Computing 2020, Vienna, Austria) using the poLCA package.^27^

## RESULTS

**Figure 1:**
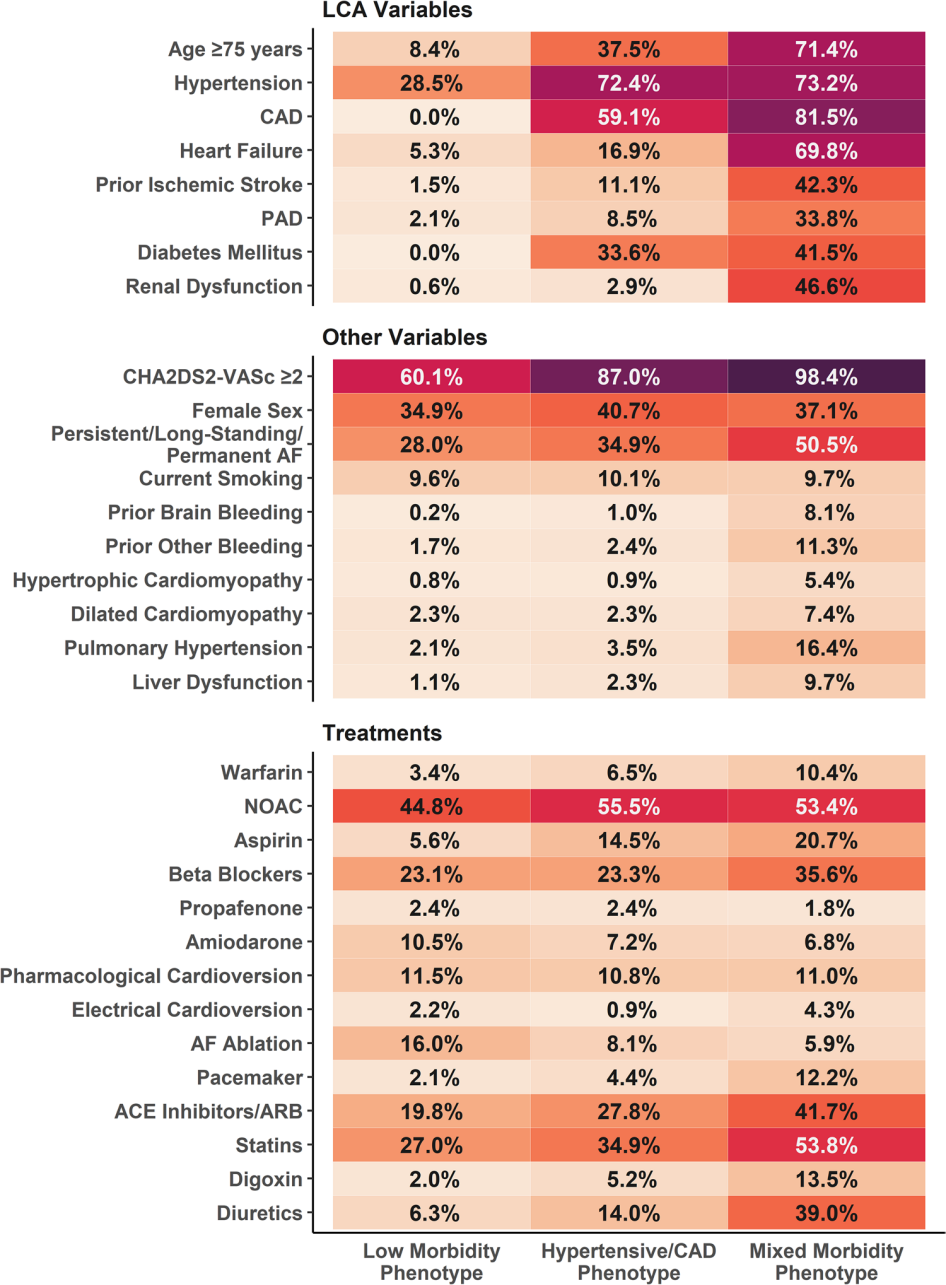
Prevalence of clinical characteristics and treatments according to the phenotypes of AF patients. ACE, angiotensin-converting enzyme; ARB, angiotensin receptor blockers; CAD, coronary artery disease; NOAC, non-vitamin K antagonist oral anticoagulant; PAD, peripheral artery disease.

A total of 3324 AF patients (mean age 68.5 ± 13.9 years; 38.0% females) were enrolled in the trial and included in the analysis. Metrics for LCA models from two to five classes are reported in Table S1 in Supplementary Materials; according to metrics and clinical judgment, the model yielding three classes were finally selected. We therefore identified three phenotypes of patients, according to the risk factors and comorbidities representation in each class (Fig. 1): (i) low morbidity phenotype (*n* = 1234, 37.1% of total patients, mean age 60.2 ± 13.2 years); (ii) hypertensive/CAD phenotype (*n* = 1534, 46.2% of total patients, mean age 71.2 ± 11.7 years); and (iii) mixed morbidity phenotype (*n* = 556, 16.7%, mean age 79.6 ± 9.8). Overall, subjects in the mixed morbidity phenotype were older, with the highest prevalence of CKD (46.6%), DM (41.5%) and history of IS (42.3%).

Baseline characteristics of the population according to mAFA allocation and phenotype are shown in Table 1; a graphical representation of the most relevant characteristics, along with treatments, is shown in Fig. 1. Among patients in the low morbidity phenotype, those allocated to mAFA were younger, and with numerically lower prevalence of several comorbidities, such as hypertension and HF. Conversely, patients in the hypertensive/CAD phenotype allocated to mAFA intervention showed less prevalence of previous IS compared to those allocated to usual care (7.6% vs 14.0%), but a higher prevalence of DM (36.9% vs. 30.9%). Finally, patients in the mixed morbidity phenotype allocated to mAFA intervention presented overall higher prevalence of several comorbidities, including hypertension, HF, DM and prior IS compared to the usual care group. Consistently, they had a higher CHA_2_DS_2-_VASc score compared to patients allocated to the usual care group.

**Table 1:** Baseline Characteristics

	Low morbidity phenotype			Hypertensive/CAD phenotype			Mixed morbidity phenotype		
	mAFA	Usual Care	*p*	mAFA	Usual Care	*p*	mAFA	Usual Care	*p*
	686	548		697	837		263	293	
Age (mean (SD))	58.16 (13.77)	62.85 (12.01)	** < 0.001**	70.80 (11.53)	71.51 (11.79)	0.233	79.23 (10.04)	79.91 (9.58)	0.416
Female, *n* (%)	214 (31.2)	217 (39.6)	**0.003**	297 (42.6)	328 (39.2)	0.191	114 (43.3)	92 (31.4)	**0.005**
Comorbidities, *n* (%)									
Smoking status	69 (10.1)	49 (8.9)	0.572	69 (9.9)	86 (10.3)	0.875	21 (8.0)	33 (11.3)	0.246
Hypertension	182 (26.5)	170 (31.0)	0.094	514 (73.7)	597 (71.3)	0.318	212 (80.6)	195 (66.6)	** < 0.001**
HF at baseline	30 (4.4)	36 (6.6)	0.115	127 (18.2)	133 (15.9)	0.253	203 (77.2)	185 (63.1)	** < 0.001**
CAD	0 (0.0)	0 (0.0)	NaN	413 (59.3)	493 (58.9)	0.930	222 (84.4)	231 (78.8)	0.114
PAD	16 (2.3)	10 (1.8)	0.676	68 (9.8)	62 (7.4)	0.121	88 (33.5)	100 (34.1)	0.939
Prior ischemic stroke	6 (0.9)	12 (2.2)	0.094	53 (7.6)	117 (14.0)	** < 0.001**	132 (50.2)	103 (35.2)	** < 0.001**
Diabetes mellitus	0 (0.0)	0 (0.0)	NaN	257 (36.9)	259 (30.9)	0.017	124 (47.1)	107 (36.5)	**0.014**
Renal dysfunction	2 (0.3)	5 (0.9)	0.288	22 (3.2)	22 (2.6)	0.643	114 (43.3)	145 (49.5)	0.172
Pulmonary hypertension	15 (2.2)	11 (2.0)	0.985	25 (3.6)	28 (3.3)	0.906	47 (17.9)	44 (15.0)	0.428
Liver dysfunction	11 (1.6)	2 (0.4)	0.066	15 (2.2)	21 (2.5)	0.772	29 (11.0)	25 (8.5)	0.396
Prior thromboembolism	10 (1.5)	7 (1.3)	0.981	14 (2.0)	23 (2.7)	0.440	30 (11.4)	29 (9.9)	0.661
Prior other bleeding	12 (1.7)	9 (1.6)	1.000	14 (2.0)	23 (2.7)	0.440	28 (10.6)	35 (11.9)	0.727
Dilated cardiomyopathy	12 (1.7)	16 (2.9)	0.238	17 (2.4)	19 (2.3)	0.961	15 (5.7)	26 (8.9)	0.206
Hyperthyroidism	14 (2.0)	11 (2.0)	1.000	10 (1.4)	19 (2.3)	0.314	13 (4.9)	21 (7.2)	0.360
Hypertrophic cardiomyopathy	6 (0.9)	4 (0.7)	1.000	8 (1.1)	6 (0.7)	0.539	11 (4.2)	19 (6.5)	0.312
Prior brain bleeding	0 (0.0)	2 (0.4)	0.384	3 (0.4)	12 (1.4)	0.084	21 (8.0)	24 (8.2)	1.000
AF type, *n* (%)			** < 0.001**			** < 0.001**			0.158
Unknown	167 (24.6)	46 (8.4)		95 (13.7)	52 (6.2)		19 (7.3)	15 (5.1)	
New-onset AF	90 (13.3)	105 (19.2)		85 (12.3)	104 (12.4)		20 (7.6)	23 (7.8)	
Paroxysmal AF	268 (39.5)	207 (37.8)		309 (44.6)	351 (42.0)		96 (36.6)	102 (34.8)	
Persistent AF	126 (18.6)	150 (27.4)		167 (24.1)	216 (25.8)		87 (33.2)	82 (28.0)	
Long-standing AF	16 (2.4)	18 (3.3)		23 (3.3)	53 (6.3)		17 (6.5)	30 (10.2)	
Permanent AF	11 (1.6)	22 (4.0)		14 (2.0)	60 (7.2)		23 (8.8)	41 (14.0)	
Prior AF treatment, *n* (%)									
Pharamcological cardioversion	104 (15.2)	38 (6.9)	< 0.001	77 (11.0)	88 (10.5)	0.800	32 (12.2)	29 (9.9)	0.472
Electrical cardioversion	18 (2.6)	9 (1.6)	0.329	4 (0.6)	10 (1.2)	0.316	8 (3.0)	16 (5.5)	0.233
AF ablation	117 (17.1)	81 (14.8)	0.316	55 (7.9)	70 (8.4)	0.808	11 (4.2)	22 (7.5)	0.140
Pacemaker	10 (1.5)	16 (2.9)	0.115	32 (4.6)	35 (4.2)	0.791	34 (12.9)	34 (11.6)	0.729
LAAO	10 (1.5)	5 (0.9)	0.544	10 (1.4)	11 (1.3)	1.000	13 (4.9)	14 (4.8)	1.000
Scores									
CHA2DS2-VASc (median [IQR])	2 [1, 3]	2 [1, 3]	0.157	3 [2, 4]	3 [2, 4]	0.125	5 [4, 6]	4 [3, 5]	** < 0.001**
HAS-BLED (median [IQR])	1 [0, 1]	1 [0, 2]	** < 0.001**	2 [1, 2]	1 [1, 2]	0.413	3 [2, 3]	2 [2, 3]	0.073
Total follow-up time (median [IQR])	260.0 [114.0, 395.0]	341.0 [239.0, 395.0]	** < 0.001**	395.0 [139.0, 395.0]	328.0 [206.0, 395.0]	0.462	278.0 [150.0, 395.0]	294.0 [158.0, 395.0]	0.464

*AF* atrial fibrillation, *CAD* coronary artery disease, *PAD* peripheral artery disease, *IQR* interquartile range, *LAAO* left atrial appendage occlusion, *PAD* peripheral artery disease, *SD* standard deviation

Treatments according to phenotypes and mAFA intervention are reported in Supplementary Table S2. Overall, use of NOAC and beta-blockers were higher in patients allocated to mAFA intervention, across all phenotypes; more intense treatment of cardiovascular comorbidities was also observed in the mAFA intervention-allocated patients, and overall, in the hypertensive/CAD and mixed morbidity phenotypes.

### Risk of Adverse Events Across Phenotypes

**Figure 2:**
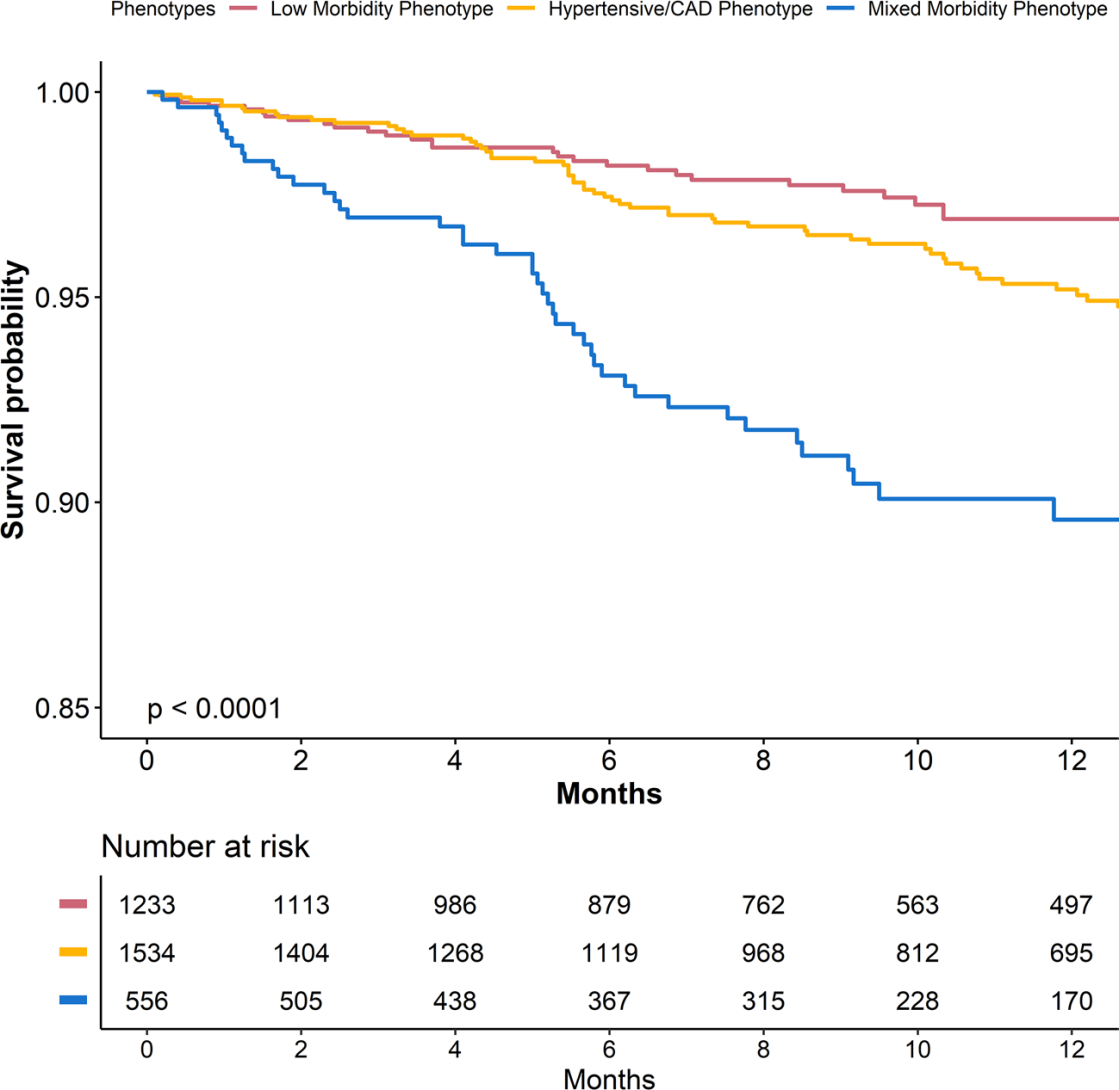
Survival analysis according to class allocation for the primary outcome.

Survival curves for the primary composite outcome according to the phenotype of AF patients are shown in Fig. 2. After a median follow-up of 295 (IQR, 152–395) days, patients in the mixed morbidity phenotype showed a low survival probability compared to the other two phenotypes (*p* < 0.001 for both). On multiple-adjusted Cox regression analysis, mixed morbidity phenotype was associated with higher hazard of the primary composite outcome (aHR [95% CI], 1.83 [1.05–3.20], Table 2) compared to low morbidity phenotype; no statistically significant results were observed for the hypertensive/CAD phenotype (aHR [95% CI], 0.98 [0.60–1.58]). Among secondary outcomes, no statistically significant differences were observed for all-cause death, while the mixed morbidity phenotype was associated with increased rehospitalisations during follow-up (aHR [95% CI], 2.45 [1.25–4.78], Table 2).

**Table 2:** Cox regression Analysis and Incidence Rate According to the Different Phenotypes

	Event Count	IR per 100 person-years (95% CI)	aHR (95% CI)	*p*
**Composite outcome of IS/TE, death and rehospitalisation**				
Low morbidity phenotype	29/1234	3.2 [2.2–4.6]	Ref.^a^	Ref
Hypertensive/CAD phenotype	57/1534	4.9 [3.7–6.3]	0.98 (0.60–1.58)^a^	0.918
Mixed morbidity phenotype	47/556	12.4 [9.1–16.5]	**1.83 (1.05**–**3.20)**^**a**^	**0.034**
**Ischemic stroke**				
Low morbidity phenotype	1/1234	0.1 [0.0–0.6]	1.00 (1.00–1.00)^a^	Ref
Hypertensive/CAD phenotype	2/1534	0.2 [0.0–0.6]	1.11 (0.09–13.77)^a^	0.937
Mixed morbidity phenotype	3/556	0.7 [0.2–2.2]	5.45 (0.39–75.29)^a^	0.206
**All-cause death**				
Low morbidity phenotype	10/1234	1.1 [0.5–2.0]	Ref.^a^	Ref
Hypertensive/CAD phenotype	15/1534	1.3 [0.7–2.1]	0.61 (0.25–1.49)^a^	0.278
Mixed morbidity phenotype	12/556	3.0 [1.6–5.3]	0.82 (0.29–2.33)^a^	0.704
**Thromboembolism**				
Low morbidity phenotype	2/1234	0.2 [0.0–0.8]	1.00 (1.00–1.00)^a^	Ref
Hypertensive/CAD phenotype	3/1534	0.3 [0.1–0.7]	0.56 (0.08–3.79)^a^	0.554
Mixed morbidity phenotype	8/556	2.0 [0.9–3.9]	2.58 (0.40–16.74)^a^	0.320
**Rehospitalisation**				
Low morbidity phenotype	18/1234	2.0 [1.2–3.1]	Ref.^a^	Ref
Hypertensive/CAD phenotype	40/1534	3.4 [2.4–4.6]	1.11 (0.61–2.00)^a^	0.737
Mixed morbidity phenotype	37/556	9.7 [6.8–13.3]	**2.45 (1.25**–**4.78)**^**a**^	**0.009**
**Bleeding (intracranial/extracranial)**				
Low morbidity phenotype	17/1234	1.9 [1.1–3.0]	Ref.^a^	Ref
Hypertensive/CAD phenotype	33/1534	2.8 [1.9–3.9]	1.65 (0.88–3.10)^a^	0.122
Mixed morbidity phenotype	19/556	4.8 [2.9–7.5]	**3.18 (1.44**–**6.98)**^**a**^	**0.004**
**Composite of recurrent AF, heart failure and ACS**				
Low morbidity phenotype	36/1234	4.0 [2.8–5.5]	Ref.^a^	Ref
Hypertensive/CAD phenotype	48/1534	4.1 [3.0–5.4]	1.00 (0.63–1.58)^a^	0.991
Mixed morbidity phenotype	45/556	11.7 [8.6–15.7]	**3.04 (1.78**–**5.19)**^**a**^	** < 0.001**

^a^Adjusted for mAFA group, sex, age, type of AF and cluster effect

### Effect of mAFA Intervention Across Phenotypes

**Figure 3:**
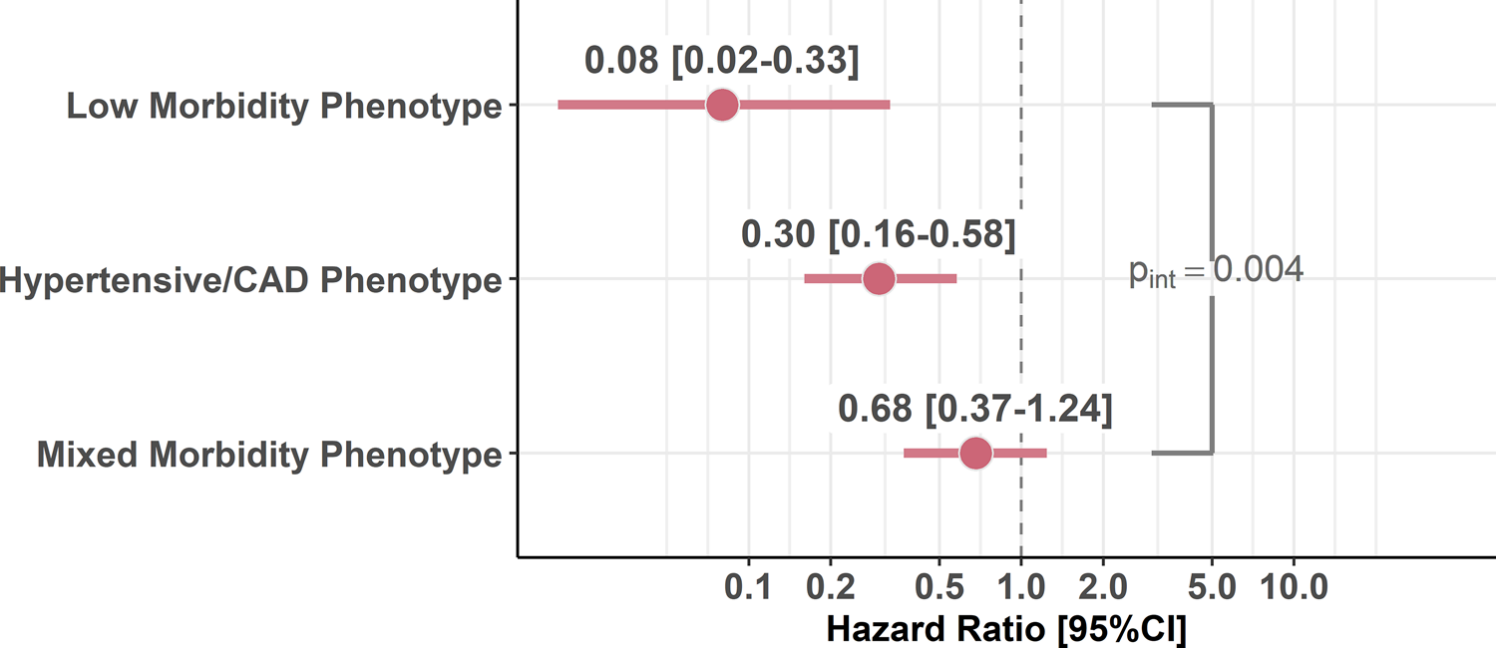
Cox-regression model for the interaction between mAFA intervention and multimorbidity class for the primary outcome. CAD, coronary artery diseases; CI, confidence intervals; *p*_int_, *p* for interaction. Adjusted for age, sex, type of AF, arterial hypertension, diabetes mellitus, CAD, heart failure, renal dysfunction, history of ischemic stroke, peripheral artery disease and cluster effect.

Results of the interaction analysis between mAFA intervention and phenotypes are reported in Fig. 3. A statistically significant interaction was observed for the effect of mAFA intervention across phenotypes of patients with AF on the risk of the primary outcome, with diluted effect as the complexity of phenotypes increased (*p* for interaction [*p*_int_] = 0.004). Specifically, the effect of mAFA intervention was highest in magnitude in the in the low morbidity phenotype (aHR, 0.08; 95% CI 0.02–0.33), while lower risk reduction was observed in the mixed morbidity phenotype (aHR, 0.68; 95% CI 0.37–1.24). Similar results were observed when analysing the interaction between mAFA intervention, phenotypes and risk of secondary outcomes (all-cause death and rehospitalisations, *p*_int=_0.088 and *p*_int=_0.020, respectively; Figure S1).

## DISCUSSION

In this post hoc analysis of the mAFA-II cluster randomised trial, our principal findings are as follows: (a) we identified three major phenotypes of patients using LCA, according to their risk factors, with increasing complexity and risk of adverse outcomes; (b) the mixed morbidity phenotype was associated with high risk of primary composite outcome compared to low morbidity phenotype; similar results were observed when considering rehospitalisations alone; and (c) mAFA intervention was associated with improved prognosis in AF patients, but with a diluted effect as the complexity of phenotype increased, and particularly in the mixed morbidity phenotype. Similar results were observed for the exploratory secondary outcomes.

In this analysis, taking into account eight of the most common comorbidities found in patients with AF, we show how different phenotypes of patients can be identified, and how patients risk profile influences the risk of major outcomes, especially among those with a more complex phenotype. These results are in accordance with previous observations from observational registries,^6,13,28,29^ and confirm that prognosis in patients with AF is strongly influenced by the underlying complexity of the clinical risk profile. Of note, in our analysis, patients in the mixed morbidity phenotype had almost fourfold higher incidence rate for the primary composite outcome, and threefold higher incidence rate of all-cause mortality compared to patient in the low morbidity phenotype; however, after covariate adjustment, we did not find a significant association for all-cause death, suggesting that age had a significant effect on the risk of death in these patients.

Our results show that a mobile health implemented ABC pathway improves prognosis in AF patients, although with diluted effects as the complexity of clinical phenotype increases. Nonetheless, in our analysis, mAFA intervention was still associated with some reduction of the risk of the primary composite outcome, suggesting that while the magnitude of the effect is conditional to the underlying complexity of the patients, an integrated care approach is still able to ameliorate prognosis in AF patients with complex phenotypes.

These results expand previous evidence on the effect of the ABC pathway in complex AF patients, such as those with multimorbidity (defined as ≥ 2 chronic conditions),^18^ and in elderly patients.^30^ Noteworthy, a similar diluted effect of the mAFA intervention was observed in very elderly AF patients,^30^ suggesting that the overall baseline risk exerts some modulating effect on the efficacy of the ABC pathway in this cohort. Indeed, we observed some differences in terms of baseline characteristics among patients allocated to mAFA intervention vs. usual care across different phenotypes; specifically, patients in the mixed morbidity phenotype allocated to mAFA intervention had higher prevalences of cardiovascular comorbidities, such as hypertension, HF, prior IS and DM; conversely, subjects allocated to mAFA intervention in the low morbidity phenotype had lower prevalences of several risk factors, compared to patients treated as per usual care. While such differences were perhaps expected due to the randomised cluster trial design, these could have influenced the results observed, and specifically, the diluted effect of mAFA intervention in the more complex phenotypes.

One previous post hoc analysis of the mAFA-II trial showed a diluted effect of the mAFA intervention in elderly patients (and particularly in those ≥ 80 years).^30^ In this analysis, we expand these observations, which were limited to an age-dependent categorisation of patients; indeed, when we included other risk factors, along with age, we observed similar results. Of note, the mean age of patients in the mixed morbidity phenotype was 79.6 years, suggesting that age may have a significant role in influencing the risk of outcomes in these patients, and therefore in diluting the effect of mAFA intervention.

Our results expand previous research, which focused on the clustering of AF patients. These investigations were mostly based on observational registries, and mainly identified different comorbidities’ clusters, which were associated with heterogeneous risks of major outcomes.^5,6,14,15,31^ These epidemiological data were important to identify and recognise the multimorbidity patterns which were at high risk of adverse events. Moreover, the identification of these patterns has also emphasised a tendency of under prescription of OAC in AF patients with multimorbidity,^3^ contributing to explain the high risk of adverse events in these individuals.

Nonetheless, in our analysis, we included both baseline comorbidities and age to define our phenotypes of AF patients. This approach is more adherent to the concept of ‘clinical complexity’, which was proposed in previous studies and that consider not only multimorbidity, but also the contribution of chronological ageing and frailty.^9,13,32^ Indeed, frailty increases with ageing and with multimorbidity, as suggested by a recent meta-analysis, and increasing frailty is implicated in the high risk of stroke, major bleeding and all-cause death.^28,29,33^ While we were unable to assess and consider frailty in this study, this may represent an explanation for our results, as one can hypothesise that patients in the more complex phenotypes had higher degree of frailty.

Indeed, the ABC pathway implemented in the mAFA-II trial was not specifically developed to manage frailty and, therefore, further studies are needed to confirm these hypotheses, and to analyse whether an approach incorporating routine assessment and management of frailty may further improve prognosis of AF patients. The undergoing AFFIRMO programme will shed light on these issues and may provide useful and definitive answers to these open questions.^34^

Along with the results on the primary outcome, we also observed a significant interaction between the patients’ phenotypes and the effect of mAFA intervention on the exploratory secondary outcomes of bleeding events and the composite outcome of non-fatal cardiovascular events, with similar results observed for the risk of all-cause death. These results, however, should be regarded as hypothesis generating, as the analysis may have been underpowered to detect differences between groups. Nonetheless, the overall higher baseline risk of events in the more complex phenotypes, along with the higher burden of comorbidities in mAFA intervention group, especially for the mixed morbidity phenotype, may explain these results.

Taken together, the results of our analysis have important clinical implications. The identification of phenotypes of AF patients with more complex patterns of risk factors is pivotal to identify those subjects for whom a more intensive management of comorbidities is needed in order to achieve better prognosis. While the ABC pathway has been developed to streamline a holistic—yet pragmatic—approach to the treatment of AF patients, our results suggest that more complex phenotypes of AF patients may require further efforts to control their overall risk of adverse events, with specific strategies aimed at managing non-cardiovascular comorbidities, and also taking into account frailty and the effects of chronological ageing. It is important to emphasise that in addition to the purely interventional aspect and optimisation of medical therapy, psychological aspects, which are only rarely considered in the approach to multimorbid and complex patients, are crucial. Improvement of care of such complex patients has to include psychological aspects and the bond created between patient and physician which is difficult to be measure quantitatively but can become better with the use of digital health technology, thus contributing to improve outcomes.

Individualisation of care, in this scenario, seems crucial to achieve better prognosis, and may require tailored intervention to increase patient compliance and to tackle the potential detrimental effects of polypharmacy.^35–37^ In this setting, and particularly among older patients, digital technology (e.g. smartphone applications) may be employed to support the awareness, although we need further evidence on the efficacy of these tools in individuals who may have difficulties in the use of digital devices, as well as cognitive and visual impairment which may represent barriers to the adoption of technology.^38,39^

### Strengths and Limitations

This study is the first to evaluate the efficacy of a mHealth-integrated ABC pathway across different phenotypes of AF patients, as identified by the combination of several baseline risk-factors. The cluster randomised trial setting in which this analysis was performed further contributes to the reliability of our results.

Nonetheless, we acknowledge some limitations. This was a post hoc analysis and there were some imbalances in the baseline characteristics among classes according to the trial allocation (mAFA intervention vs. usual care). These imbalances might have influenced our results. Moreover, this analysis might be underpowered for some comparisons performed. Additionally, even if we performed a Cox-regression analysis adjusted for several factors, we cannot exclude the contribution of other potential moderators or residual confounders; frailty and social determinants of health, particularly, are known to exert a significant role in influencing the natural history of patients with AF,^33,40–42^ and it is likely that these factors disproportionally affected patients with more complex phenotypes. Moreover, although we applied multiple adjusted Cox-regression analysis, other approaches and models have been proposed for use with latent class analyses,^43^ which may yield different results; therefore, our results should be interpreted with some caution. Further studies are required to investigate the influence of these factors on both the characterisation of the patterns of AF patients, as well as on the effectiveness of integrated care approaches in this clinical setting. Our results on secondary outcomes should be regarded as exploratory and hypothesis-generating, as these were not adjusted for multiple comparisons, and considering the relatively low rates for some events.

## CONCLUSIONS

In this ancillary analysis of the mAFA-II trial, the ABC pathway improves prognosis in patients with AF across different comorbidity phenotypes, although with some differences in the magnitude of risk reduction. Patients with more complex phenotypes may require further efforts to improve their outcomes, considering their high baseline risk of adverse events.

## Supplementary Information

Below is the link to the electronic supplementary material.

Supplementary file1 (DOCX 83 KB)

## Data Availability

Data supporting the current study are available from the corresponding author on reasonable request.
