# Supplementary material for: Efficacy of the ABC Pathway for Integrated Care Across Phenotypes of Patients with Atrial Fibrillation: A Latent-Class Analysis Report from the mAFA-II Clinical Trial
Source: J Gen Intern Med. 2024 Oct 28;40(6):1238–47. doi: 10.1007/s11606-024-09037-6 (PMC12045915; doi:10.1007/s11606-024-09037-6)
Supplement: Supplementary file 1 — Supplementary file1 (DOCX 83 KB) [file 11606_2024_9037_MOESM1_ESM.docx]

**Efficacy of the ABC pathway for integrated care across phenotypes of patients with Atrial Fibrillation: A latent-class analysis report from the mAFA-II clinical trial**

Supplementary Materials

**MAFA II investigators**

**Executive Steering Committee:**

| Yutao Guo | Chinese PLA General Hospital, Beijing, China (Co-Chair) |
| --- | --- |
| Gregory Y H Lip | Institute of Cardiovascular Sciences, University of Birmingham, UK; Liverpool Centre for Cardiovascular Science, University of Liverpool, UK (Co-chair) |
| Deirdre A. Lane | Liverpool Centre for Cardiovascular Science, University of Liverpool, UK |
| Yundai Chen | Chinese PLA General Hospital, Beijing, China |
| Liming Wang | The National Center for Chronic and Noncommunicable Disease Control and Prevention, Chinese Center for Disease Control and Prevention, Beijing, China |

**Steering committee:**

| Jens Eckstein | University Hospital Basel, Switzerland |
| --- | --- |
| G Neil Thomas | Institute of Applied Health Research, University of Birmingham, United Kingdom |
| Liu Tong | The Second Hospital of Tianjin Medical University, Tianjin, China |
| Feng Mei | Shanxi Dayi Hospital, Taiyuan, Shanxi, China |
| Liu Xuejun | Affiliated First Hospital, Shanxi Medical University, China |
| Li Xiaoming | Cardiovascular Disease Hospital of Shanxi Province, China |
| Shan Zhaoliang | PLA General Hospital, Beijing, China |
| Shi Xiangming | PLA General Hospital, Beijing, China |
| Zhang Wei | PLA Army General Hospital, Beijing, China |
| Xing Yunli | Beijing Friendship Hospital, Capital Medical University, Beijing, China |
| Wen Jing | Beijing Haidian Hospital, Beijing, China |
| Wu Fan | Tianjin Medical University General Hospital, Tianjing, China |
| Yang Sitong | The First Affiliated Hospital, Ji Lin University, Ji Lin, China |
| Jin Xiaoqing | Tongji Hospital, Tongji medical College, Huazhong University Of Science & Technology, Wuhan, China |
| Yang Bo | Xiangya Hospital Central South University, Changsha, China |
| Bai Xiaojuan | ShengJing Hospital of China Medical University, Shengyang, China |
| Jiang Yuting | Suqian Hospital, Jiangsu, China |
| Liu Yangxia | General Hospital of Shengyang Military, Shengyang, China |
| Song Yingying | Bozhou Renmin Hospital, Anhui, China |
| Tan Zhongju | The First Hospital of Zhejiang Province, Hangzhou, China |
| Yang Li | Yunnan Cardiovascular Hospital, Kunming, China |
| Luan Tianzhu | The First Affiliated Hospital of Haerbing Medical University, Haerbing, China |
| Niu Chunfeng | The Second Affiliated Hospital of Haerbing Medical University, Haerbing, China |
| Zhang Lili | The Fourth Affiliated Hospital of Haerbing Medical University, Haerbing, China |
| Li Shuyan | The First Affiliated Hospital, Ji Lin University, Ji Lin, China |
| Wang Zulu | General Hospital of Shengyang Military, Shengyang, China |
| Xv Bing | The First People's Hospital of Shengyang, Shengyang, China |
| Liu Liming | The Second Affiliated Hospital of Shengyang Medical University, Shengyang, China |
| Jin Yuanzhe | The Fourth Affiliated Hospital of China Medical University, Shengyang, China |
| Xia Yunlong | The First Affiliated Hospital of Dalian Medical University, Dalian, China |
| Chen Xiaohong | The People's Hospital of Liaoning Province, Shengyang, China |
| Wu Fang | Rui Jin Hospital, Tong university School of Medicine, Shanghai, China |
| Zhong Lina | The Affiliated Hospital of Qingdao University, Qingdao, China |
| Sun Yihong | China-Japan Friendship Hospital, Beijing, China |
| Jia Shujie | Beijing Anzhen Hospital, Capital Medical University, Beijing, China |
| Li Jing | Xuanwu Hospital Capital Medical University, Beijing, China |
| Li Nan | The Third People’s Hospital of Dalian, Dalian, China |
| Li Shijun | Dalian Municipal Central Hospital Affiliated of Dalian Medical University, Dalian, China |
| Liu Huixia | Guangdong Academy of Medical Sciences Guangdong General Hospital, Guangdong, China |
| Li Rong | The First Affiliated Hospital of Guangzhou University of Traditional Chinese Medicine, Guangzhou, China |
| Liu Fan | The Second Hospital of Hebei Medical University, Hebei, China |
| Ge Qingfeng | North China University Science And Technology Affiliated Hospital |
| Guan Tianyun | The Second Hospital of Jilin University, Jilin, China |
| Wen Yuan | The Second Affiliated Hospital of Nanchang University, Nanchang, China |
| Li Xin | BenQ Hospital affiliated to Nanjing Medical University, Nanjing, China |
| Ren Yan | Ruijin Hospital, Shanghai Jiao Tong University School of Medicine |
| Chen Xiaoping | Taiyuan City Central Hospital, Taiyuan, China |
| Chen Ronghua | Tangshan People's Hospital, Tangshan, China |
| Shi Yun | Tianjin Fourth Central Hospital , Tianjin, China |
| Zhao Yulan | The Second Affiliated Hospital of Zhengzhou University, Zhengzhou, China |
| Shi Haili | Zhengzhou Central Hospital Affiliated to Zhengzhou University, Zhengzhou, China |
| Zhao Yujie | Zhengzhou Seventh People's Hospital, Zhengzhou, China |
| Wang Quanchun | Shenyang Fifth People's Hospital, Shenyang, China |
| Sun Weidong | Taian City Central Hospital, Taian, China |
| Wei Lin | Harbin First Hospital, Harbin, China |

**Data Safety Monitoring Board:**

| Esther Chan | The University of Hong Kong, Hong Kong, China |
| --- | --- |
| Shan Guangliang | Department of Epidemiology and Statistics, Institute of Basic Medical Sciences, Peking Union Medical College, Beijing, China |
| Yao Chen | Peking University Clinical Research Institute, Beijing, China |
| Zong Wei | China Foreign Affairs University, Beijing, China |
| Chen Dandi | West China School of Public Health, Chengdu, China |

**Clinical events committee:**

| Han Xiang | Department of Neurology, Huashan Hospital of Fudan University, Shanghai, China |
| --- | --- |
| Xu Anding | Department of Neurology, the First Affiliated Hospital of Jinan University, Guang Zhou, China |
| Fan Xiaohan | Fuwai Hospital, Chinese Academy of Medical Sciences, Beijing, China |
| Yu Ziqiang | Institute of Blood Research of Jiangsu Province, China |
| Gu Xiang | Department of Cardiology, People’s Hospital of Subei, Affiliated Hospital of YangZhou University, Jiangsu Province, China |
| Ge Fulin | Department of Gastroenterology, Chine PLA General Hospital, Beijing, China |

**SUPPLEMENTARY TABLES**

**Supplementary Table 1 – Metrics for Latent Class Models**

| **Number of Classes** | **BIC** | **cAIC** | **Percent size of the smallest class** |
| --- | --- | --- | --- |
| 2 Classes | 25974.85 | 25991.85 | 37.9% |
| **3 Classes** | **25914.57** | **25940.57** | **16.7%** |
| 4 Classes | 25938.22 | 25973.22 | 12.1% |
| 5 Classes | 25970.90 | 26014.90 | 0.5% |

Legend: BIC: Bayesian Information Criteria; cAIC: consistent Akaike Information Criteria

**Supplementary Table S2. Baseline treatments according to mAFA allocation and multimorbidity phenotypes.**

|  | **Low Morbidity Phenotype** | | | **Hypertensive/CAD Phenotype** | | | **Mixed Morbidity Phenotype** | | |
| --- | --- | --- | --- | --- | --- | --- | --- | --- | --- |
|  | **mAFA** | **Control** | **p** | **mAFA** | **Control** | **p** | **mAFA** | **Control** | **p** |
| n | 686 | 548 |  | 697 | 837 |  | 263 | 293 |  |
| **Pathway A** | | | | | | | | | |
| Warfarin | 12 (1.7) | 30 (5.5) | **0.001** | 23 (3.3) | 76 (9.1) | **<0.001** | 20 (7.6) | 38 (13.0) | 0.054 |
| NOACs | 384 (56.0) | 169 (30.8) | **<0.001** | 483 (69.3) | 368 (44.0) | **<0.001** | 166 (63.1) | 131 (44.7) | **<0.001** |
| Aspirin | 17 (2.5) | 52 (9.5) | **<0.001** | 71 (10.2) | 151 (18.0) | **<0.001** | 41 (15.6) | 74 (25.3) | **0.007** |
| Clopidogrel | 11 (1.6) | 11 (2.0) | 0.752 | 39 (5.6) | 82 (9.8) | 0.003 | 36 (13.7) | 36 (12.3) | 0.715 |
| Ticagrelor | 1 (0.1) | 4 (0.7) | 0.248 | 5 (0.7) | 16 (1.9) | 0.074 | 3 (1.1) | 6 (2.0) | 0.610 |
| **Pathway B** | | | | | | | | | |
| Beta-Blockers | 209 (30.5) | 76 (13.9) | **<0.001** | 193 (27.7) | 164 (19.6) | **<0.001** | 116 (44.1) | 82 (28.0) | **<0.001** |
| Propafenone | 15 (2.2) | 15 (2.7) | 0.661 | 13 (1.9) | 24 (2.9) | 0.268 | 2 (0.8) | 8 (2.7) | 0.154 |
| Amiodarone | 111 (16.2) | 19 (3.5) | **<0.001** | 64 (9.2) | 47 (5.6) | **0.010** | 25 (9.5) | 13 (4.4) | **0.028** |
| **Pathway C** | | | | | | | | | |
| ACE Inhibitors | 50 (7.3) | 43 (7.8) | 0.794 | 103 (14.8) | 112 (13.4) | 0.477 | 36 (13.7) | 63 (21.5) | **0.022** |
| ARB | 99 (14.4) | 49 (8.9) | **0.004** | 111 (15.9) | 112 (13.4) | 0.182 | 78 (29.7) | 66 (22.5) | 0.069 |
| Diuretics | 26 (3.8) | 52 (9.5) | **<0.001** | 76 (10.9) | 139 (16.6) | **0.002** | 102 (38.8) | 115 (39.2) | 0.980 |
| Calcium Channel Blockers | 52 (7.6) | 28 (5.1) | 0.102 | 177 (25.4) | 136 (16.2) | **<0.001** | 69 (26.2) | 65 (22.2) | 0.310 |
| Statins | 203 (29.6) | 130 (23.7) | **0.025** | 270 (38.7) | 266 (31.8) | **0.005** | 150 (57.0) | 149 (50.9) | 0.169 |
| Digoxin | 12 (1.7) | 13 (2.4) | 0.570 | 42 (6.0) | 38 (4.5) | 0.235 | 39 (14.8) | 36 (12.3) | 0.452 |
| Nitrate | 6 (0.9) | 14 (2.6) | 0.036 | 116 (16.6) | 129 (15.4) | 0.558 | 70 (26.6) | 95 (32.4) |  |

**Legend:** ACEi= Angiotensin converting enzyme inhibitor, ARB= Angiotensin receptor blockers, CCB= Calcium Channel Blockers; DM= Diabetes Mellitus; NOACs= Non-vitamin K Oral Anticoagulants. Hypoglycemic Agents: at least one among sulfonylurea, biguanide, alpha glucosidase inhibitors and other agents.

**SUPPLEMENTARY FIGURES**

**Supplementary Figure S1. Cox-regression model for the interaction between mAFA intervention and multimorbidity class for secondary outcomes.**


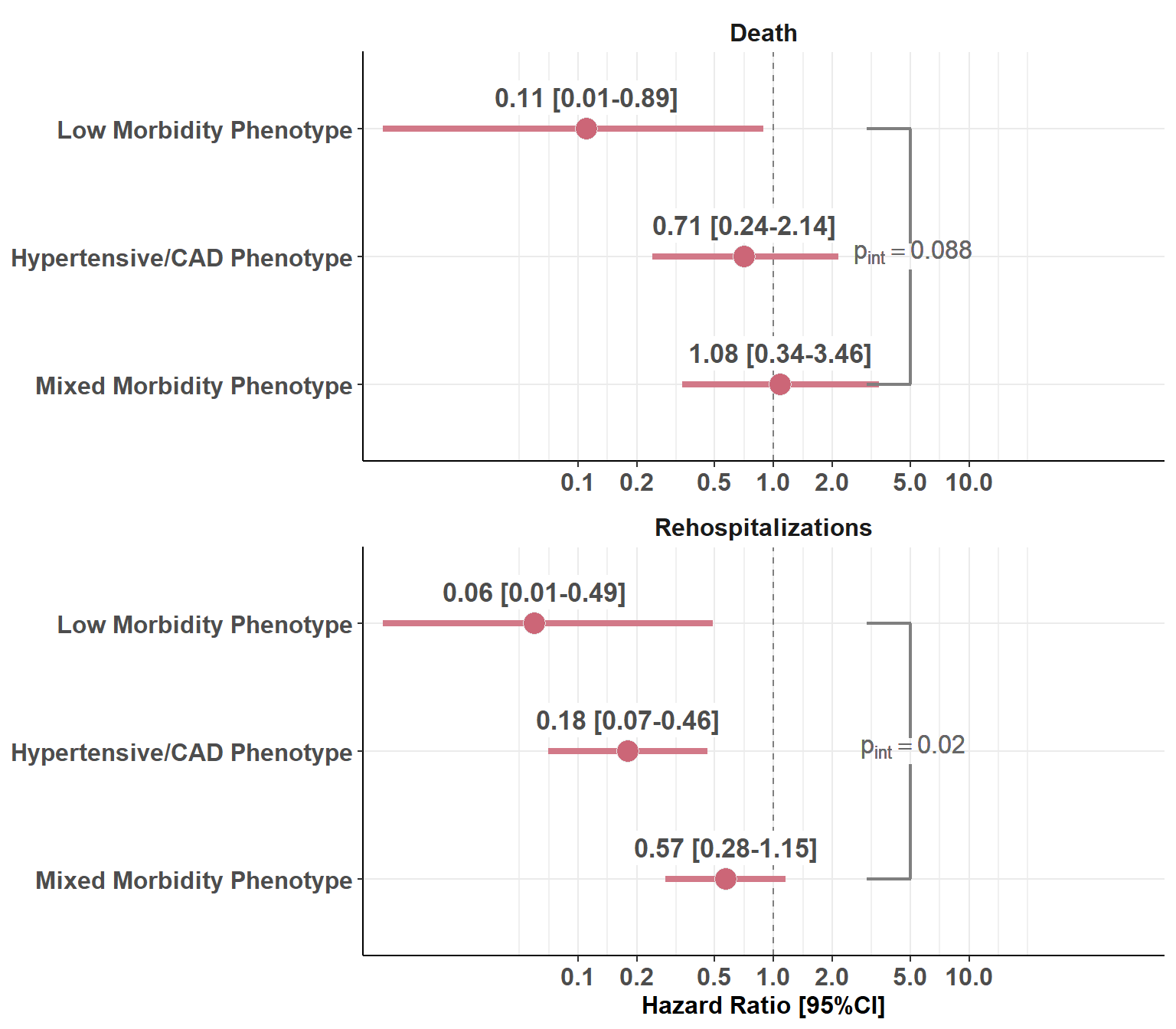


**Legend:** CAD= Coronary Artery Diseases; CI = Confidence Intervals; p_int_= p for interaction. Adjusted for age, sex, type of AF, arterial hypertension, diabetes mellitus, CAD, heart failure, renal dysfunction, history of ischemic stroke, peripheral artery disease and cluster effect.
